# Long-Term Outcomes of the Restoration of Uterovaginal Continuity and Vaginoplasty—Utero-Colo-Neovaginoplasty—in Cervicovaginal Agenesis Using the Sigmoid Colon

**DOI:** 10.1007/s00192-024-05878-1

**Published:** 2024-07-25

**Authors:** Vijay Kumar, Sundeep Payyanur Thotan, Santosh P Prabhu, Pratap Kumar Narayan, Nitin G Pai, Ranjani Rammohan

**Affiliations:** 1 Department of Pediatric Surgery, Kasturba Medical College, Manipal, Manipal Academy of Higher Education, Manipal, Karnataka 576104 India; 2 Department of Reproductive Medicine and Surgery, Kasturba Medical College, Manipal, Manipal Academy of Higher Education, Manipal, 576104 India

**Keywords:** Cervicovaginal agenesis, Vaginal agenesis, Utero-colo-neovaginoplasty, Sigmoid vaginoplasty, Hematometra

## Abstract

**Introduction and Hypothesis:**

Congenital cervicovaginal agenesis in the presence of a functional endometrium is a rare Müllerian anomaly. The management ranges from hysterectomy historically to various reconstructive procedures more recently. We report our experience with utero-colo-vaginoplasty in the management of this anomaly and its long-term follow-up.

**Methods:**

The case records of all the patients with vaginal or cervicovaginal agenesis in our hospital from January 2002 to December 2019 were reviewed retrospectively. The patients were then called for an outpatient visit and examined in detail. The anatomical variations, surgical procedures and outcomes were recorded and analysed.

**Results:**

Sixteen patients aged 14 to 26 years were included during the study period. They presented with cyclical painful cryptomenorrhea. Magnetic resonance imaging (MRI) confirmed cervicovaginal or distal vaginal agenesis. All the patients underwent utero-colo-vaginoplasty. Intraoperative rectal injury led to post-operative faecal leak from the perineal wound in one patient in the post-operative period. Restoration of painless menstrual flow was possible in all 16 cases. Long-term complications were seen in 4 patients. These were stenosis of the perineal neovaginal orifice in 2 patients, obstruction at colo-uterine anastomosis in 1 patient and mucosal prolapse at the neovagina in 1 patient. Three of these patients needed secondary surgical procedures. Five were sexually active and reported consummation of penetrative intercourse. None of them had conceived.

**Conclusion:**

In our experience, utero-colo-vaginoplasty allows for regular painless menstruation and coitus with minimal long-term complications. The sole disadvantage is the failure to conceive.

## Introduction

Vaginal and cervicovaginal agenesis are uncommon Müllerian malformations [1]. The fallopian tubes, ovaries and uterus are normal in these patients. The recommended treatments in the past included hysterectomy, cervical canalisation and various reconstructive procedures, as described by different authors [2–4]. However, some of the uterus-preserving operations have shown significant long-term complications [5–7]. Some of the patients are not willing to undergo hysterectomy for social and cultural reasons. They need the construction of a conduit for both menstruation and sexual function without significant long-term morbidity. Our patients underwent utero-colo-neovaginoplasty as described by Kannaiyan et al. [7] using the sigmoid colon as a conduit, the distal end of which is used for construction of the neovagina, and the proximal end is anastomosed to the posterior wall of the uterus or to the upper vaginal pouch. In this study, we review the immediate and long-term outcomes of this reconstruction at our centre.

## Materials and Methods

The in-patient and outpatient medical records of all patients with vaginal or cervicovaginal agenesis in our hospital between January 2002 and December 2019 were reviewed and data collected retrospectively. Patients with other Müllerian and similar reproductive anomalies who chose other therapies were excluded from this study. From each patient’s record, we collected data regarding demographics, clinical features, imaging investigations, anatomical variations in the anomaly, associated anomalies, type of surgery performed and complications, both immediate and late. Follow-up data for outcomes were obtained from outpatient records, outpatient visits and telephone interviews. They were asked about ease of menstrual flow, abdominal pain and excess mucous discharge from the neovagina. Married women were interviewed along with their partners with regard to coital satisfaction. Ethical clearance was obtained from the Institutional Ethics Committee. There was no involvement of the patients or public in the design, conduct, reporting, or dissemination plans of the study.

**Fig. 1:**
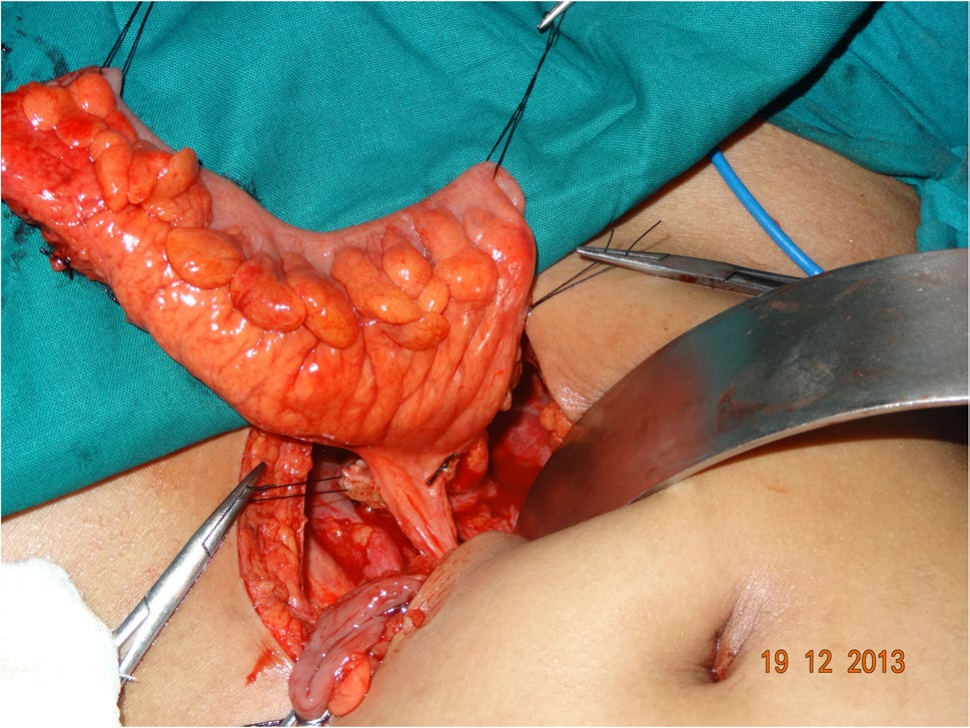
Isolated sigmoid colon segment with vascular pedicle

**Fig. 2:**
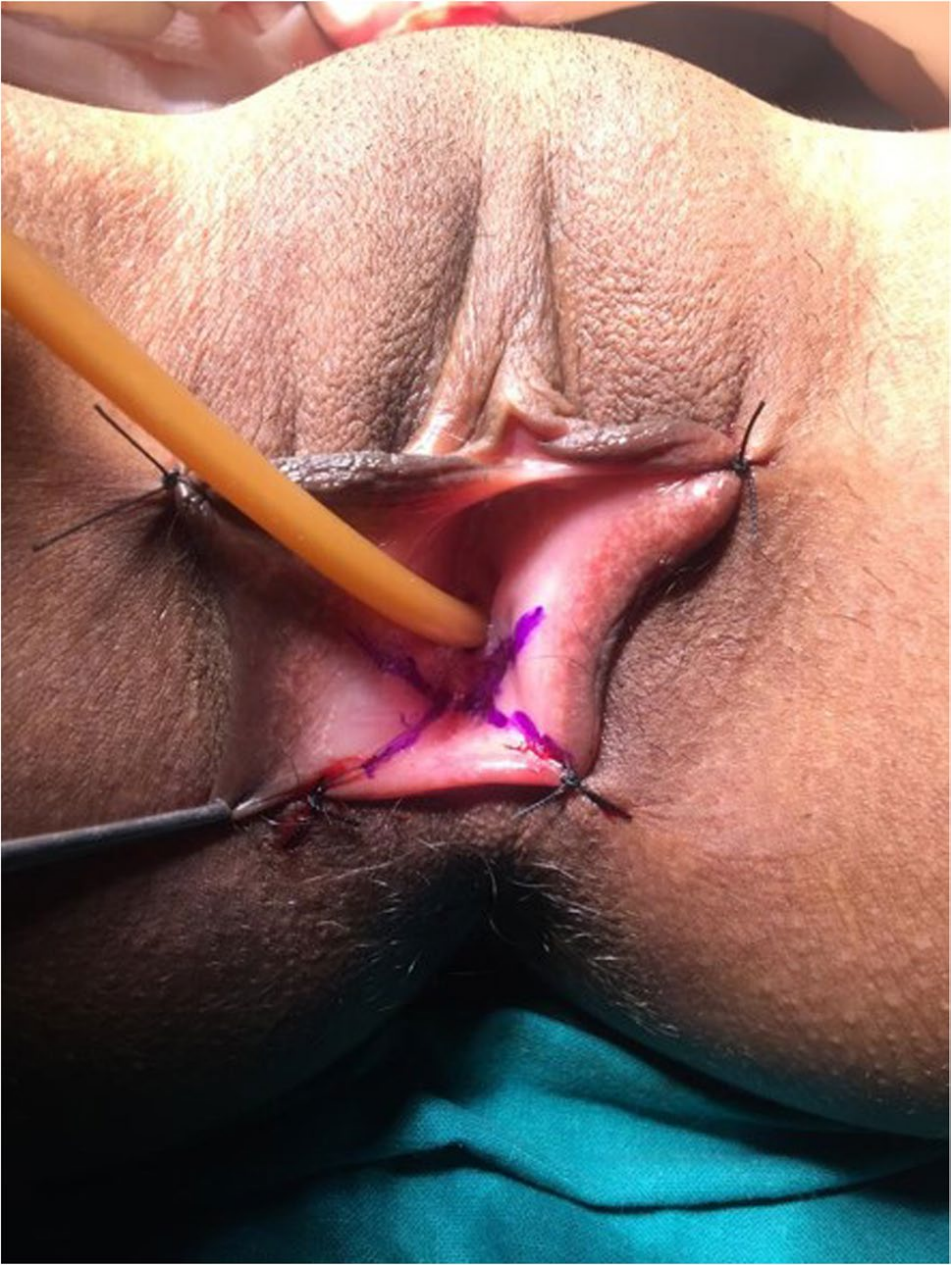
Cruciate incision in the vestibule to develop vaginal mucosal flaps

**Fig. 3:**
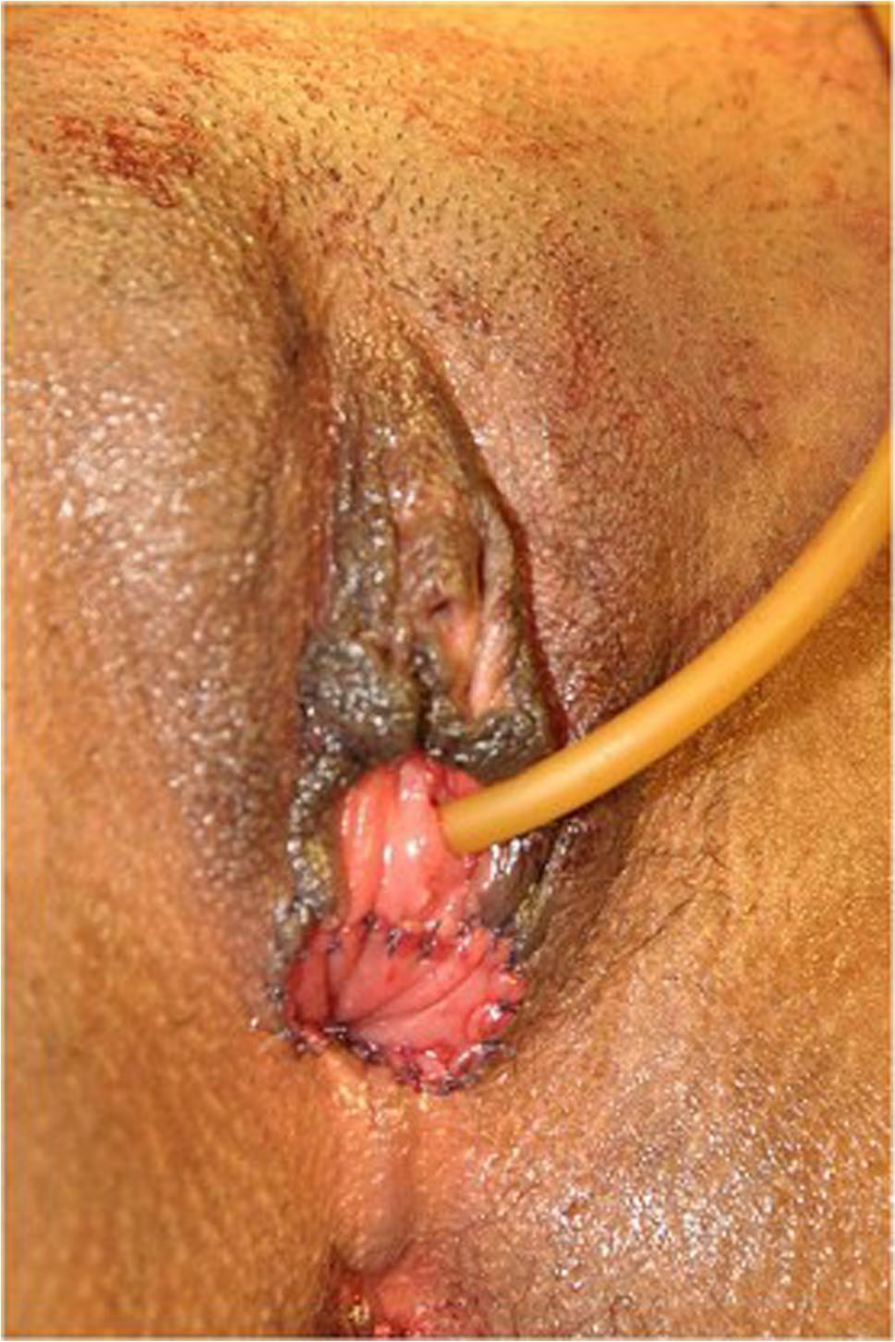
Neovaginal orifice after vaginoplasty

**Fig. 4:**
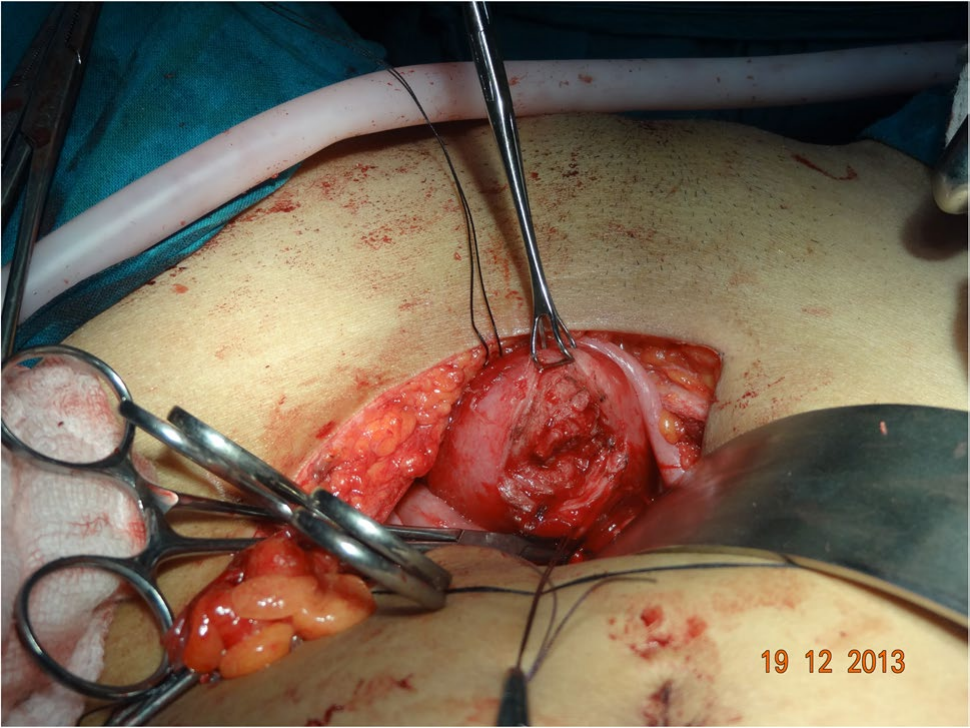
Wide opening on the posterior wall of the uterus for uterus-to-colon anastomosis

All patients underwent vaginal reconstruction and creation of a uterovaginal conduit using a segment of the sigmoid colon. The procedure begins with a laparotomy through a Pfannenstiel incision. After an assessment of the pelvic structures, a 10- to 15-cm segment of the sigmoid colon with its vascular pedicle is isolated (Fig. 1). Bowel continuity is restored by anastomosing the proximal and distal ends of the colon using a handsewn method. Next, on the perineal end, an X-shaped cruciate incision is marked in the vaginal vestibule with the centre at the vaginal pit or where the vagina should be (Fig. 2). Four V-shaped mucocutaneous flaps are made in the vestibule from the X-shaped incision. A space is created using blunt dissection posterior to the catheterised urethra and anterior to the rectum. A finger or gauze pack is placed in the rectum to guide the dissection. As the dissection reaches the peritoneal reflection, it is incised from the abdominal end to create a tunnel for the colon conduit to the perineum. The space is serially dilated using Hegar’s dilators and the sigmoid colon conduit is brought down through the space. If the length of the distal end of the conduit is not able to reach the perineum comfortably for anastomosis, the conduit can be rotated 180° so that the proximal end can be brought to the perineum for tension-free anastomosis. The lower end of the colon conduit is sutured to mucocutaneous flaps in an interdigitating fashion using 3–0 polyglactin (Vicryl, Ethicon) sutures (Fig. 3). In patients with cervicovaginal agenesis, the upper end of the colon was anastomosed to the posterior wall of the uterus after performing a circular myomectomy of 3–4 cm using 3–0 polyglactin (Vicryl, Ethicon) (Fig. 4). In patients with an upper vaginal pouch, the upper end of the sigmoid colon conduit was anastomosed to the vaginal pouch. A drain was placed in the peritoneal cavity and the abdomen closed in layers. Oral feeds were started on the 3rd post-operative day. The urinary catheter was removed on the 5th day and the patient was discharged on the 7th day after surgery. The first follow-up was done after 1 month. Subsequent visits were scheduled for 3, 6, 12 months and then annual follow-ups.

## Results

**Fig. 5:**
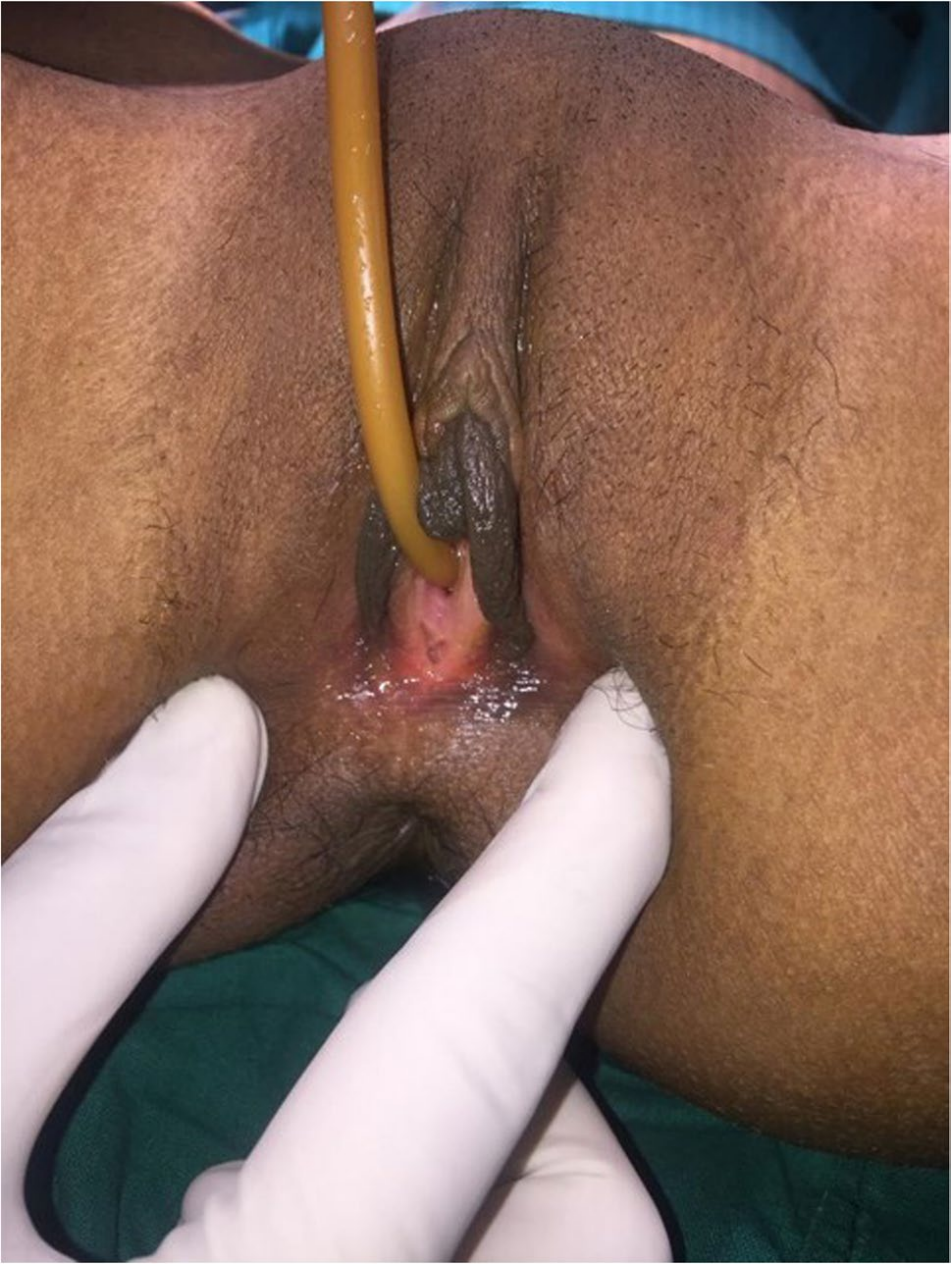
Absent vaginal orifice

**Fig. 6:**
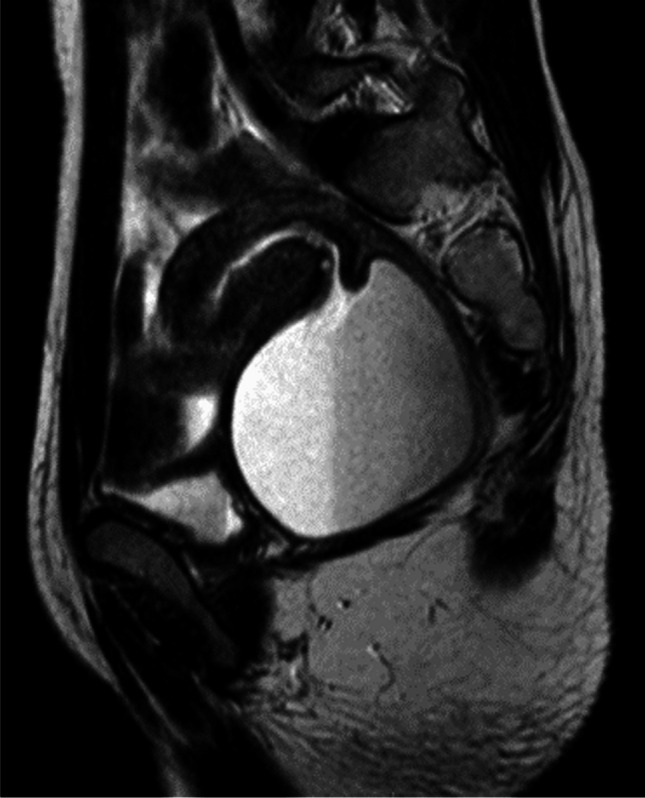
Magnetic resonance imaging showing haematocolpos in the proximal vagina

We obtained the records of 16 patients in the period between 2002 and 2019 with a diagnosis of vaginal or cervicovaginal agenesis. They were in the age group ranging from 14 to 26 years (median age 18.3 years). All of them presented with primary amenorrhea and cyclical abdominal pain. The average duration of symptoms was 4.5 years. Most of the patients were from a rural background, without early and easy access to tertiary care health care facilities. Prior to referral to our centre, they had been managed symptomatically using analgesics, traditional ayurvedic medications and some hormonal preparations. Eight patients (53%) had undergone surgical procedures before reaching our centre, which included perineal exploration (2), hymenectomy (1), salpingectomy (2), anoplasty (1), diagnostic laparoscopy (1), laparotomy and uterotomy (1), and McIndoe’s vaginoplasty (1). The secondary sexual characteristics were normal in all patients. The external appearance of the genitalia was similar to that of a normal female with an absent vaginal orifice in 13 cases (Fig. 5), a short, blind-ending vaginal pit in 2 cases and a stenosed orifice with scarring in the patient who had undergone a prior unsuccessful vaginoplasty. Ultrasound and magnetic resonance imaging (MRI) of the abdomen and pelvis confirmed the absence of the cervix and vagina, with haematometra and/or haematocolpos (Fig. 6). Twelve patients had cervicovaginal agenesis and four had agenesis of the distal vagina. Four of the patients with cervicovaginal agenesis also had a bicornuate uterus. There were associated extra-genital anomalies in some patients. Two patients had an anterior ectopic anus. One patient had left renal agenesis. Two of the patients had ovarian cysts and 1 patient had endometriosis (Table 1).

**Table 1:** Clinical and treatment details of patients

Sl. no.	Age (years)	Presentation	Genital tract anomalies ASRM type	Extragenital anomalies	Previous surgeries (before referral)	Surgical treatment	Follow-up/marital status	Marital status	Complications
1	14	Cyclical abdominal pain and primary amenorrhoea for 2 years	Cervicovaginal agenesis, left unicornuate uterus with distal left atrophic uterine remnant, left ovarian cystadenofibroma	Nil	Nil	Left ovarian cystectomy, excision of the left horn of the uterus, sigmoid vaginoplasty	8 years	Not married	Stenosis of colouterine anastomosis
2	22	Cyclical abdominal pain for 9 years	Distal vaginal agenesis, haemorrhagic cyst in the right ovary	Left renal agenesis	Nil	Sigmoid vaginoplasty	10 years	Married	Nil
3	16	Cyclical abdominal pain for 3 years and primary amenorrhoea	Cervicovaginal agenesis, left unicornuate uterus with distal left atrophic uterine remnant, multiloculated left ovarian cyst, haemorrhagic cyst in the right ovary	Left ectopic pelvic kidney	Appendicectomy (4 years back), left salpingectomy (2 years back)	Left ovarian cystectomy, excision of the left horn of the uterus and sigmoid vaginoplasty	10 years	Not married	Neovaginal stenosis at the perineum (responded to dilatation)
4	22	Cyclical abdominal pain for 9 years and primary amenorrhoea	Cervicovaginal agenesis, endometriosis	Nil	Nil	Sigmoid vaginoplasty	10 years	Married	Nil
5	26	Dysmenorrhoea	Cervicovaginal agenesis, septate uterus	Nil	McIndoe’s vaginoplasty 3 years back	Undoing of McIndoe’s vaginoplasty, metroplasty, sigmoid vaginoplasty	8 years	Married	Neovaginal stenosis at the perineum (underwent Y-V plasty)
6	20	Cyclical abdominal pain for 5 years and primary amenorrhoea	Cervicovaginal agenesis	Perineal fistula as anterior ectopic anus, anorectal malformation	Nil	Anterior sagittal anorectoplasty, sigmoid vaginoplasty	9 years	Not married	Rectal injury and faecal fistula in the perineum (responded to conservative treatment)
7	16	Cyclical abdominal pain and primary amenorrhoea for 3 years	Distal vaginal agenesis	Left renal agenesis	Perineal exploration	Sigmoid vaginoplasty	10 years	Not married	Neovaginal prolapse (underwent excision of the prolapsed segment)
8	17	Cyclical abdominal pain and primary amenorrhoea for 4 years	Cervicovaginal agenesis, bicornuate uterus with bilateral obstructed endometrial cavities, endometriosis	Nil	Nil	Sigmoid vaginoplasty	10 years	Not married	Nil
9	22	Cyclical abdominal pain and primary amenorrhoea for 6 years	Cervicovaginal agenesis, right tubo-ovarian complex mass, endometriosis	Nil	Laparotomy and uterotomy	Right ovarian cystectomy, sigmoid vaginoplasty	10 years	Married	Nil
10	16	Cyclical abdominal pain and amenorrhoea for 3 years	Cervicovaginal agenesis	Left renal agenesis	Nil	Sigmoid vaginoplasty	11 years	Not married	Nil
11	14	Cyclical abdominal pain and primary amenorrhoea for 2 years	Distal vaginal agenesis	Nil	Nil	Sigmoid vaginoplasty	12 years	Not married	Nil
12	15	Cyclical abdominal pain and primary amenorrhoea for 3 years	Distal vaginal agenesis	Nil	Diagnostic laparoscopy	Sigmoid vaginoplasty	9 years	Not married	Nil
13	14	Cyclical abdominal pain and primary amenorrhoea for 3 months	Cervicovaginal agenesis	Anterior ectopic anus	Anoplasty	Anterior sagittal anorectoplasty, sigmoid vaginoplasty, colostomy	11 years	Not married	Nil
14	15	Cyclical abdominal pain abdomen and primary amenorrhoea for 3 years	Cervicovaginal agenesis, bicornuate uterus with atretic right horn	Nil	Right salpingectomy (6 months back)	Excision of the right horn and sigmoid vaginoplasty	8 years	Not married	Nil
15	26	14 years of painful irregular periods and intermittent lower abdominal pain. Difficulty with sexual intercourse for 4 months	Cervicovaginal agenesis, bilateral haematosalpinx	Nil	Hymenectomy and perineal exploration, vaginal dilatation 5 times under general anaesthetic	Excision of the stenotic vagina and sigmoid vaginoplasty	9 years	Married	Nil
16	16	Primary amenorrhoea and cyclical abdominal pain for 3 years	Cervicovaginal agenesis	Nil	Nil	Sigmoid vaginoplasty	2 years	Not married	Nil

All patients and their parents or spouses underwent counselling by a team of paediatric surgeons and gynaecologists before the surgery. Reproductive implications were discussed and documented. The first author was the primary surgeon in all cases. The mean operating time was 236 ± 30 min and the average blood loss was 300 ± 50 ml. In four patients with distal vaginal agenesis, the upper end of the sigmoid colon conduit was anastomosed to the upper vaginal pouch. In 12 patients with cervicovaginal agenesis, the upper end of the conduit was anastomosed to the posterior wall of the uterus.

Some of the patients required additional procedures. Of the four patients with bicornuate uterus, three of them had atretic horns on one side (right—1, left—2). The atretic horns were excised in the same session. The two patients with an anterior ectopic anus underwent simultaneous anterior sagittal anorectoplasty (ASARP). A covering colostomy was also performed along with ASARP in one patient. Three of the patients with ovarian cysts underwent a cystectomy. The stenotic vagina in the patient who had undergone a McIndoe’s vaginoplasty earlier had to be excised prior to a sigmoid vaginoplasty.

There was one surgical complication. One patient had an accidental rectal injury during creation of the space between the rectum and urethra. The tear was closed primarily; however, she developed minimal faecal discharge from the perineal wound. She responded to the conservative line of management before discharge from the hospital.

At discharge, all patients were put on hormonal treatment to delay the next menstrual cycle. Hormonal therapy was continued if there were features of endometriosis. Patients were counselled regarding excessive postoperative vaginal discharge and advised to perform gentle saline washouts to keep the perineum clean.

The follow-up period ranged from 2 to 12 years with a mean follow-up of 9 years post-surgery. All patients had the first follow-up at 1 month after the surgery. At subsequent follow-up visits, 4 out of 16 patients developed complications. The rest resumed a painless cyclical menstrual flow. Two patients developed stenosis at the neovaginal orifice. One patient had a mucosal prolapse at the neovaginal orifice. One patient developed obstruction at the proximal anastomosis between the colon and uterus, which was managed by revision anastomosis.

Of the 2 patients who had stenosis of the neovaginal orifice, 1 responded to regular self-dilatation. The other patient did not respond to self-dilatation. She was managed with a V-Y plasty of the neovaginal orifice. The mucosal prolapse was managed by excision of the prolapsed portion of the conduit. Thus, 3 of the 4 patients who had complications required a second surgery, although, in 2 patients, the second surgery was minor. None of these patients had any long-term effects (Table 1).

At long-term follow-up, all 16 patients were menstruating regularly, and there was no dysmenorrhea. None of the patients had excessive mucous discharge requiring usage of sanitary pads. Five patients were sexually active with male partners and reported satisfactory intercourse. All 5 were interviewed along with their partners and had satisfactory coital function.

All the patients were counselled in detail regarding the feasibility of reproduction and documented. Four of our patients with a normal uterus and cervix with an upper vaginal pouch were advised to proceed with pregnancy after consulting their obstetrician but cautioned against vaginal delivery, although none had achieved pregnancy by the last follow-up.

## Discussion

Congenital cervicovaginal agenesis is due to defective development in the Müllerian ducts and sinovaginal bulbs. The failure of distal Müllerian structures to develop may give rise to agenesis of the cervix and proximal vagina. Failure of the development of the sinovaginal bulbs and fusion with the Müllerian duct can cause agenesis of the lower part of the vagina with an intact cervix and a blind-ending upper part of the vagina [8]. The uterus, tubes, ovaries and external genitalia are normal in this clinical condition, unlike Mayer–Rokitansky–Kuster–Hauser syndrome, where there are varying degrees of uterine hypoplasia ranging from rudimentary uterus to uterine agenesis. Congenital agenesis of the uterine cervix and vagina in the presence of a functional endometrium is an extremely rare Müllerian anomaly. The incidence of congenital vaginal atresia is reportedly 1 in 4,000 to 1 in 5,000 female births, with normal development of the uterus observed in less than 10% of these subjects [9]. The American Society of Reproductive Medicine (ASRM) system of classification categorises vaginal agenesis as 1a and cervicovaginal atresia as 1b [9, 10]. ASRM classification is widely used. The other classification systems for Müllerian anomalies are the embryological–clinical classification [11], the Vagina Cervix Uterus Adnexa-associated Malformation classification [12] and the European Society of Human Reproduction and Embryology/European Society for Gynaecological Endoscopy classification [13].

Patients with cervicovaginal agenesis present with primary amenorrhea and cyclical pain in the abdomen as they have a functioning endometrium. Generally, these anomalies go undiagnosed until sometime after menarche, as these patients have age-appropriate female secondary sexual characteristics, and the external genitalia may look normal [2]. Pain is due to obstructed menstrual flow or endometriosis. The primary goals of treatment in these patients are to construct a conduit for a free flow of menstrual blood, thereby relieving cyclical pain and preserving the uterus; a neovagina for sexual intercourse and, if possible, to maintain fertility. Historically, these groups of patients were managed using different procedures such as hysterectomy and neovaginoplasty, cervical canalisation combined with vaginoplasty or uterovaginal/uterovestibular anastomosis [13–18]. There are limited data available on the safety and success of various reconstructive surgical procedures [18]. Rock et al. [14] and Fujimoto et al. [2], in their review, have reported high restenosis rates with recanalisation procedures. A failed procedure with inadequate menstrual drainage would cause pyometra [7].

The patients in our series have a poor socio-economic status and a rural background where adequate medical facilities are not always available. The average duration of symptoms in our series was 4.5 years, which indicates misdiagnosis and improper guidance or referral. Approximately half (53%) of our patients underwent failed surgeries before reaching our centre, resulting in scarring. Apart from relieving pain, preservation of the uterus and menstruation is also a social and cultural affirmation of gender role identity in this set of women. The absence of a uterus in a woman and the resultant infertility would have a huge negative influence on the chances of the woman being accepted in an arranged marriage practice prevalent in a conservative Indian rural background. The ability to afford the increased cost of multiple/staged procedures becomes difficult for these patients. With this background, we decided on restoration of uterovaginal continuity by utero-colo-neovaginoplasty/sigmoid colon vaginoplasty as the ideal procedure to fulfil the treatment aims of pain relief, preservation of the uterus, restoration of menstrual flow and the ability to have sexual intercourse. Although Kannaiyan et al. [7] described this surgery as a “compromise” in view of unphysiological anastomosis between the colon and uterus at the cost of compromised fertility, we still feel that, with a follow-up of 2 to 12 years (average 9 years) without much morbidity, this procedure can be considered in this set of patients with health care limitations.

There were four (25%) complications in our series in the initial couple of patients. Two (12.5%) developed stenosis of the neovaginal orifice. One (6.25%) developed stenosis of the colo-uterine anastomosis and 1 more had mucosal prolapse at the neovaginal orifice. None had an anastomotic leak. All the patients eventually had resumption of painless cyclical menstrual flow.

Similar rates of neovaginal stenosis were seen in the literature. Kannaiyan et al. [7] reported 1 (9%) stenosis in 11 patients, whereas Kisku et al. [13] reported 2 (10%) in 20 patients. Kannaiyan et al. [7] proposed a wide myomectomy by excising a 3- to 4-cm disc of the posterior wall of the uterus to avoid stenosis and obstruction at the colo-uterine anastomosis. We have adopted the same in our later cases and found it to be useful. The distal end of the sigmoid colon conduit was anastomosed to the vaginal vestibular edge in a circular end-to-end fashion initially. This was later modified as a cruciate anastomosis to the V-shaped mucosal flaps raised in the vestibule, which we believe has helped us to avoid neovaginal stenosis. Tension in vestibular anastomosis can be avoided by rotating the sigmoid conduit by 180° for anti-peristaltic anastomosis to the vestibule. Prolapse of the neovagina and excessive mucosal discharge from the sigmoid conduit can be minimised by keeping the length of the conduit optimum, as a redundant sigmoid conduit can be responsible for these complications. Tailoring of the lower segment of the sigmoid conduit by excising a linear strip of conduit in its ante-mesenteric border is done if the diameter of the conduit is quite wide. The advantage of the sigmoid conduit is that it is lubricated, lined with epithelium and resistant to local trauma. Five of our patients have male partners and are sexually active. When the couples were interviewed, they did not report any feelings of inadequacy during any stage of the sexual act.

Minimal access surgery has further improved the outlook for these complex anomalies. Alborzi et al. reported a series of 7 cases of cervicovaginal atresia in whom the sigmoid colon vaginoplasty was done laparoscopically, with good outcomes [19].

Fertility in cervicovaginal or vaginal agenesis depends on the presence or absence of the cervix. Pregnancy should be possible in the presence of a competent cervix if there are no other fertility issues. Four patients in this series with distal vaginal agenesis having a normal cervix and upper vaginal pouch were counselled to have a pregnancy but not vaginal delivery. Obstetricians should be aware of the altered anatomy and be prepared for caesarean section.

Patients with cervicovaginal agenesis have a lower chance of fertility [5, 20]. Associated uterine anomalies, endometriosis and previous attempts at recanalisation further reduce the fertility [5, 9, 13, 20]. Few successful pregnancies have been reported in the literature [3, 5, 16, 21] in the case of cervical atresia following various recanalisation procedures such as cervical drilling [15], stenting [3, 16] and skin graft [18]. Recanalisation procedures carry a higher risk of stenosis and pyometra. Additionally, compared with isolated cervical atresia, these recanalisation procedures may not be possible for cervicovaginal agenesis. The utero-colo-neovaginoplasty is a wide patent anastomosis allowing free drainage of uterine content, but, at the same time, it is associated with a high chance of pregnancy loss or related complications. We are not aware of any reports of successful pregnancy in patients with cervicovaginal agenesis who have undergone sigmoid colon vaginoplasty. Thirteen patients with cervicovaginal agenesis in our series were counselled not to conceive and opt for surrogacy. In surrogacy, the retrieval of oocytes is performed regularly via a transvaginal posterior fornix approach. This may not be possible in view of the altered anatomy in these patients, and the best possible approach will be laparoscopic transabdominal ultrasound-guided oocyte aspiration [22]. One of our patients was counselled for the same by our infertility experts.

Given the rarity of the condition, our study is limited by the number of cases. This could be addressed by a multicentre review or a systematic review with meta-analysis.

## Conclusion

Cervicovaginal or vaginal agenesis is a rare complex Müllerian anomaly with significant morbidity as well as social impact in terms of sexual function and fertility. Management can be challenging and needs to be addressed considering the socio-economic factors of the patient and family. Utero-colo-neovaginoplasty using the sigmoid colon can be considered a reliable single procedure with a satisfactory outcome to establish a reliable conduit for menstrual flow and sexual intercourse with minimal long-term complications. The sole compromise is fertility, particularly in patients with cervicovaginal agenesis.

## Data Availability

The hospital records of the patients included in the study are stored in the medical records database of our hospital. The anonymised data supporting the study are available on request to the corresponding author.
